# Analysis of Wellbore Wall Deformation in Deep Vertical Wells Based on Fiber Bragg Grating Sensing Technology

**DOI:** 10.3390/s25247626

**Published:** 2025-12-16

**Authors:** Wenchang Huang, Haibing Cai, Longfei Yang, Zixiang Li

**Affiliations:** School of Civil Engineering and Architecture, Anhui University of Science and Technology, Huainan 232001, China; 2023200272@aust.edu.cn (W.H.); longfeiyang202209@163.com (L.Y.); lzx4269016@163.com (Z.L.)

**Keywords:** shaft wall deformation, fiber Bragg grating, vertical additional stress, failure mechanism outer shaft lining

## Abstract

Accurate deformation monitoring is essential for ensuring the stability of deep vertical shafts. In this study, a temperature-compensated fiber Bragg grating (FBG) sensing system was deployed in the 882 m deep Guotun Coal Mine shaft to measure circumferential and vertical strains at six depths. A site-specific mechanical model integrating stratigraphy, dual-layer concrete lining, and the influence radius was developed to analyze shaft wall stresses. The monitoring results reveal pronounced spatial anisotropy, with circumferential compressive and tensile strains at deeper levels nearly twice those at shallow levels. Strain variation also increases over time, reflecting the combined effects of groundwater fluctuations and overburden consolidation. The stresses inferred from measured strains agree well with the analytical solution in both magnitude and depth-dependent trend, with deviations remaining within a reasonable engineering margin. All stresses are below the strength limits of the C70/C50 concrete lining, confirming that the shaft is in a safe stress state. The proposed monitoring–analysis framework provides a reliable basis for evaluating shaft wall behavior under complex hydrogeological conditions.

## 1. Introduction

The shaft of a vertical well is essential for extracting and transporting coal resources. As mining operations progress into deeper and more complex geological formations, preventing shaft damage and ensuring personnel safety are critical concerns [1,2]. Current analyses reveal that water loss from the aquifer at the bottom of the shaft leads to compression and settlement of the overlying soil layers. This process exerts additional vertical forces on the well wall, resulting in damage. As the strata settle, these additional forces on the well wall accumulate and intensify [3,4,5]. Li [6] conducted shear tests simulating the interaction between soil and the well wall, confirming that the factors influencing additional stress include shaft diameter, cross-sectional area, elastic modulus, soil layer thickness, and compression in the bottom aquifer. Zhang et al. [7] developed a mechanical model to quantify soil friction acting on the well wall, formulating a calculation for the additional pressure. Cheng et al. [8] provided a thorough analysis of mechanisms leading to sudden water influx and sand intrusion in large-diameter shafts, primarily resulting from tensile failure.

In addition to analyzing the mechanisms of wellbore failure, it is essential to monitor strain data from the wellbore and conduct timely safety assessments. Traditional wellbore monitoring methods, such as the plumb line and compressed wood methods, rely on geometric techniques to measure wellbore deformation [9]. However, these techniques have limitations, including relatively low surface measurement accuracy and a lack of real-time data transmission.

Since the beginning of this century, fiber optic technology has been increasingly adopted in the engineering field, leading to new methods for monitoring wellbore deformation [10,11,12]. These methods effectively address the shortcomings of traditional techniques. For example, Pang et al. [13] used fiber Bragg grating (FBG) sensors to monitor strain and inclination in wellbore walls. Liu et al. [14] applied Brillouin Optical Time Domain Reflectometry (BOTDR) distributed fiber optics for automated monitoring of soil deformation around the wellbore. Additionally, Zhang et al. [15] integrated distributed fiber optics with parallel electrical measurement techniques to monitor strain and displacement in thick unconsolidated layers in coal seam mining areas.

While these studies focus on the application of integrated fiber optic monitoring systems, many do not adequately consider the stratigraphic distribution of surface soil and thin bedrock. Furthermore, they often lack reasonable and detailed analyses of the monitoring results.

This study targets the auxiliary shaft of the Guotun Coal Mine in Shandong, China. It employs FBG sensors to monitor circumferential and vertical strain. The study discusses the selection and rationale for sensor placement, considering the thick unconsolidated layers and thin bedrock distribution. It analyzes the variations in circumferential and vertical strain with depth and time, elucidating the underlying mechanisms. Building on this analysis, the paper calculates the analytical solution for the vertical additional stress in the wellbore, thus verifying its safety under stress. The research findings offer a theoretical foundation for health monitoring and safety assessments of the wellbore.

## 2. Analytical Solution of Wellbore Wall Stress

**Figure 1:**
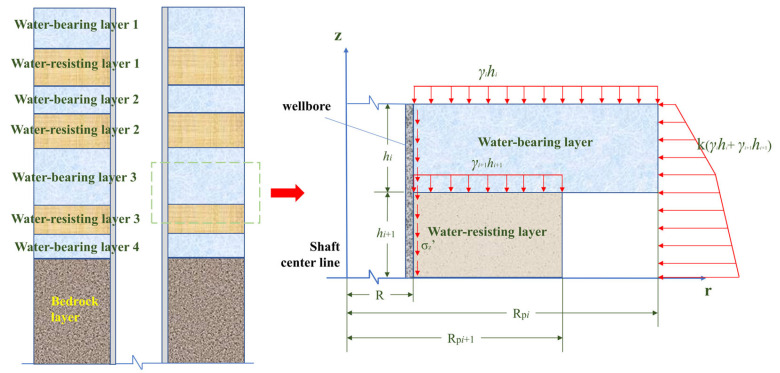
Mechanical model.

To assess the safety of the wellbore, monitoring results must be compared with theoretical values. Figure 1 displays the surrounding soil distribution around the wellbore, and a mechanical model is established using a portion of the soil as an example. In the figure, k represents the soil lateral pressure coefficient, γ is the soil unit weight, h is the soil layer thickness, and Rp is the influence radius. The following assumptions must be satisfied when calculating the vertical and circumferential stresses:

(1) Both the soil layers and the concrete wellbore are assumed to be isotropic elastic bodies, in line with the basic principles of elastic mechanics.
(2) The bedrock layer is assumed to possess high rigidity, and its settlement is considered negligible. Only the upper weathered bedrock layer and aquifer layer undergo vertical displacement.
(3) The vertical additional forces on the wellbore wall are mainly the elastic vertical force and the plastic vertical force. The plastic zone may influence the upper part of the wellbore wall, resulting from the relative displacement between surface soil settlement and the wellbore wall, which generates shear forces exceeding the elastic shear strength limit [16]. In this study, FBG sensors are placed in the lower section of the wellbore, assuming that the entire wellbore exists within an elastic shear zone.
(4) The monitoring scheme includes sensors arranged in the circumferential and vertical directions. Therefore, it is assumed that the radial deformation of the concrete wellbore wall can be disregarded, i.e., μ_r_ = 0.
(5) The range of the soil’s influence on the wellbore wall is limited by a maximum influence radius R*_pi_* [16], which is determined by the following Equation (1):
  (1) $$R_{pi}=0.5\left(1-\nu_{i}\right)l$$
  where ν*_i_* represents the Poisson’s ratio of each soil layer; *l* is the depth of the elastic segment of the wellbore, defined in this study as the distance from the upper edge of the wellbore to the corresponding soil layer.

According to the theory of thick-walled cylindrical shells in material mechanics, the circumferential stress on the inner surface of the wellbore under external pressure σ*_θ_* is given by:

(2) $$\sigma_{\theta }=\frac{2R^{2}P}{R^{2}-R_{0}^{2}}$$

where R represents the outer radius of the wellbore; R_0_ is the inner radius of the wellbore; and P is the pressure exerted by the surrounding soil on the wellbore wall, which can be calculated as kγh. In this study, the lateral pressure coefficient k is taken as 0.6.

The vertical stress σ_z_ acting on the wellbore wall can be divided into two components: one is the self-weight stress σ_c_ generated by the wellbore wall, and the other is the vertical additional stress σ_z*i*_ resulting from the friction between the soil and the wellbore wall. The self-weight stress σ_c_ can be determined by the following equation σ_c_ = γ_c_H, where γ_c_ represents the unit weight of the concrete wellbore, and H is the depth of the wellbore. The process for determining the vertical additional stress σ_z*i*_ is outlined as follows.

Based on the boundary conditions of this mechanical model, the vertical displacement function for the weathered bedrock section can be derived as:

(3) $$\omega_{i}=\omega_{i-1}+\left[1-{\left(\frac{R_{pi}-r}{R_{pi}-R}\right)}^{2}\right]\left[\frac{A_{i}}{h_{i}}\left(z-\sum_{i=1}^{n}h_{i-1}\right)+\frac{B_{i}}{h_{i}^{2}}{\left(z-\sum_{i=1}^{n}h_{i-1}\right)}^{2}\right]$$

In this equation, R is the outer radius of the wellbore, r is the distance from the centerline of the wellbore to any point in the surrounding soil, hi is the thickness of the soil layer, and A*_i_* and B*_i_* are undetermined constants. The variable ω_1_ signifies the vertical displacement of the weathered bedrock layer, specifically at the location of the sixth-level sensor, extending upwards to the first-level monitoring layer, ω_6_.

The undetermined constants are determined using the energy method from elastic mechanics. Given that the radial deformation is zero, the expression for the strain energy of an infinitesimal element simplifies to:

(4) $$u_{i}=\frac{E_{i}}{2\left(1+\nu_{i}\right)}\left[\frac{1}{2}{\left(\frac{\partial \omega_{i}}{\partial r}\right)}^{2}+2{\left(\frac{\partial \omega_{i}}{\partial z}\right)}^{2}\right]$$

where E*_i_* represents the elastic modulus of the corresponding soil layer, and ν*_i_* denotes the Poisson’s ratio of the corresponding soil layer.

The total strain potential energy *U* of a given soil layer is obtained by integration as:

(5) $$U=2\pi \int_{h_{i}}^{h_{i+1}}\int_{R}^{R_{pi}}u_{i}rdrdz$$

The work W_G*i*_ performed by the gravitational force of the soil layer is given by:

(6) $$W_{Gi}=2\pi \int_{h_{i}}^{h_{i+1}}\int_{R}^{R_{pi}}\gamma_{i}\omega_{i}rdrdz$$

where γ*_i_* represents the unit weight of the soil layer.

According to the principle of minimum potential energy, the strain potential energy U and W_G*i*_ must satisfy a specific condition given by Equation (7). Based on this principle, the expressions for the undetermined constants can be derived:

(7) $$\frac{\partial \left(U-W_{Gi}\right)}{\partial A_{i}}=\frac{\partial \left(U-W_{Gi}\right)}{\partial B_{i}}=0$$

After solving for these constants and substituting them back into Equation (3), the displacement function expressions for each soil layer can be obtained. Then, the vertical additional stress σ_z*i*_ at the wellbore wall can be determined by combining the geometric and physical equations, yielding:

(8) $$\sigma_{zi}^{\prime }=\int_{h_{i}}^{h_{i+1}}\frac{\pi RE_{i}}{1+\nu_{i}}{\left(\frac{\partial \omega_{i}}{\partial r}\right)}_{r=R}dz$$

## 3. Layout of FBG Monitoring System

### 3.1. Principle of Fiber Bragg Grating Sensing

**Figure 2:**
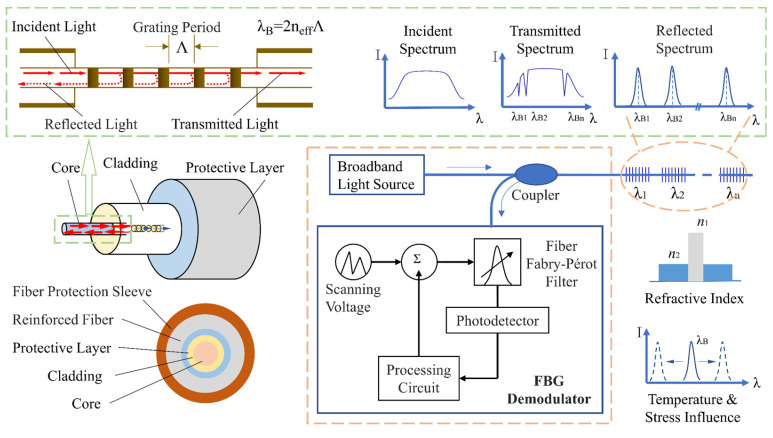
Basic principle of fiber Bragg grating.

FBG sensors are used to monitor vertical and circumferential strains of the shaft wall. These sensors offer strong environmental adaptability, including high-temperature and corrosion resistance, along with excellent transmission performance characterized by high precision and long distances [17,18]. Their wide application range makes them cost-effective and easy to package. The principle behind FBG sensors involves periodic changes in the core’s refractive index, which are influenced by temperature and stress. This causes some incident light from a broadband source to be reflected back, while the majority continues to transmit as transmitted light [19,20]. A corresponding demodulator detects variations in the reflected wavelength, allowing for the determination of changes in the measured object based on the relationship between the physical quantity and the reflected wavelength. The principle of FBG is illustrated in Figure 2.

According to the thermo-optic effect of fiber Bragg grating, the influence of temperature variation on the change in effective refractive index Δ*n*_eff_ of the FBG can be expressed as follows:

(9) $$\Delta n_{\mathrm{eff}}=\zeta \cdot n_{\mathrm{eff}}\cdot \Delta T$$

where ζ is the thermo-optic coefficient of the optical fiber. For conventional silica fibers, ζ = 6.7 × 10^−6^ °C^−1^.

The shift in the central wavelength of an FBG is jointly affected by external mechanical strain and temperature variation. Therefore, the relationship between the FBG central wavelength shift and the applied strain and temperature change can be expressed as:

(10) $$\frac{\Delta \lambda }{\lambda }=\left(1-p_{e}\right)\Delta \varepsilon +\left(\alpha_{f}+\zeta \right)\Delta T$$

where *p*_e_ is the photo-elastic coefficient of the fiber material, *p*_e_ =0.22; *α*_f_ is the thermal expansion coefficient of the fiber. For commonly used silica FBG, *α*_f_ =5.5 × 10^−7^ °C^−1^.

Consequently, two sets of FBG sensors are required to be installed in the field. One set is subjected to the combined effects of mechanical strain and temperature variation, while the other set is encapsulated within a protective tube and isolated from direct contact with the structure, thus responding only to temperature changes. This configuration enables effective temperature compensation, ensuring that the measured strain represents the true mechanical strain of the structure without interference from thermal effects.

The FBG interrogator manufactured by Suzhou Nanzee Company is used to acquire the wavelength signals from the strain sensors. It enables real-time data transmission via a 3G/4G network and operates reliably under harsh environmental conditions, featuring simple operation and rapid response. The technical specifications of the interrogator are listed in Table 1.

### 3.2. Project Profile

The auxiliary shaft of the Guotun Coal Mine is located on the western wing of the Juye syncline in southern Shandong. The area features a topography that slopes from west to east. The shaft reveals 583.1 m of Quaternary and Neogene unconsolidated strata, divided into three aquifers and two aquicludes. Beneath this, the Permian bedrock has a thickness of 292.4 m and includes two aquifers. The coal-bearing strata are primarily found in the Carboniferous Taiyuan Formation and the Permian Shanxi Formation. Geologically, the mining area exhibits high water yield from the aquifers and a significant thickness of unconsolidated strata, which gradually decreases from west to east. The auxiliary shaft has a depth of 882 m and a net diameter of 6.5 m. The upper section is constructed using a freezing method, featuring a double-layer reinforced concrete lining. Both the inner and outer walls have a thickness of 1200 mm, separated by a 3 mm plastic interlayer. The lower section employs conventional construction methods with a wall thickness of 500 mm. The upper double-layer lining consists of C70 grade concrete, which has an elastic modulus of 3.7 × 10^4^ MPa and a Poisson’s ratio of ν = 0.23. The lower plain concrete is of C50 grade.

**Figure 3:**
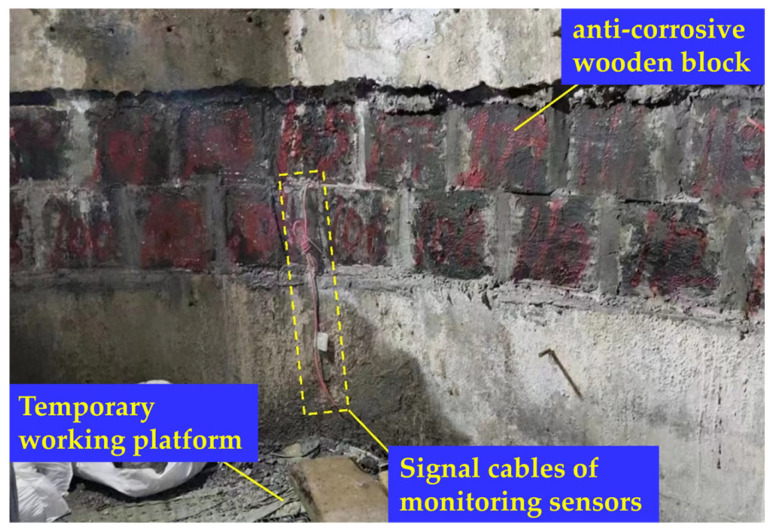
Compressible device.

Recently, the compressibility of the shaft wall has reached its limit, leading to cracking and spalling of the concrete. This poses a risk to the mine’s operational safety and necessitates urgent monitoring of shaft indicators and the implementation of remedial measures. Therefore, considering the hydrogeological conditions and the construction technique of the shaft lining, two compressible devices were installed at depths of −500 m and −565 m along the shaft. These compressible devices refer to horizontal circumferential grooves excavated in the shaft lining. Each groove has a height of 600 mm and a width of 1000 mm, and is filled with waterproof mortar and double-layer anti-corrosive wooden blocks, as illustrated in Figure 3.

Functioning as deformation-absorbing zones, these devices are designed to accommodate the vertical displacement induced by overburden settlement, thereby effectively reducing the vertical additional forces acting on the shaft lining, alleviating stress concentration, and ensuring the structural stability and long-term safety of the shaft.

### 3.3. FBG Sensor Layout Scheme

The deployment of sensors across six levels has been chosen based on several key factors: (1) The lithology of these levels primarily consists of loose sandstone, which has high water content and is susceptible to dehydration-induced deformation [21]. (2) Deformation or fractures in the wellbore are mostly found in the middle to lower sections. The second level is located beneath the first expandable device, while the fifth level is above the second expandable device. This positioning allows for accurate measurement of deformation related to both expandable devices. (3) There is significant additional stress on the wellbore at the interface between loose layers and bedrock.

**Figure 4:**
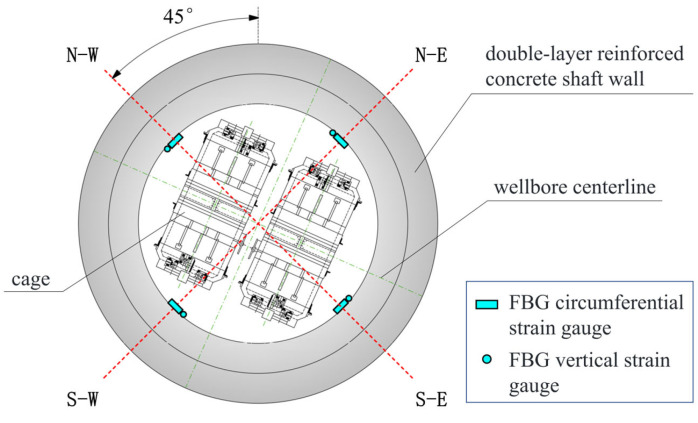
FBG layout diagram.

As a result, sensors are installed within the sandstone layers between depths of 481 m and 583 m. Table 2 outlines the specific installation levels and corresponding soil parameters. The first, second, and third levels are within the middle aquiclude of the Neogene system, while the fourth and fifth levels are in the lower aquiclude. The sixth level is situated in the weathered bedrock aquifer. Each level has eight sensors, composed of four vertical strain gauges and four circumferential strain gauges. These gauges are grouped together, with four groups installed in a cross pattern around the wellbore, facing southeast, northeast, northwest, and southwest. A schematic diagram of the sensor arrangement is shown in Figure 4.

**Figure 5:**
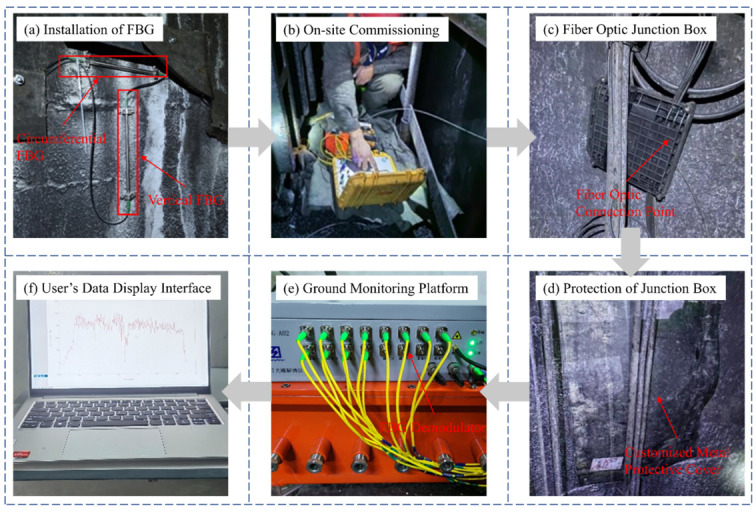
On-site Installation Steps of Monitoring System.

**Figure 6:**
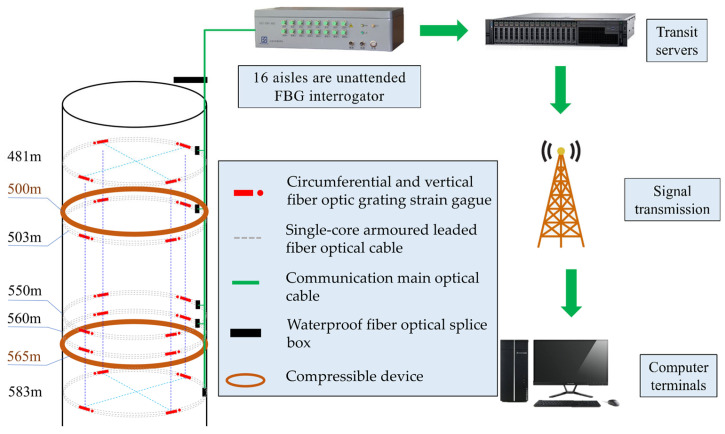
Schematic diagram of the detection system.

The installation process for fiber optic grating sensors in the field is detailed in Figure 5. Due to the complex construction environment of vertical shafts, careful routing of optical cables is critical during monitoring. Junction boxes are required at the connection points between the optical cables and the fiber optic grating sensors for protection. After the connections are completed, these junction boxes should be secured to the shaft wall and covered with custom-designed protective covers. Once the installation of the fiber optic grating sensors is complete, on-site debugging is essential to ensure proper operation before data collection at the ground monitoring platform begins. The schematic diagram of the detection system is shown in Figure 6.

## 4. Analysis of Monitoring Results

### 4.1. Relationship Between Strain and Shaft Wall Depth

**Figure 7:**
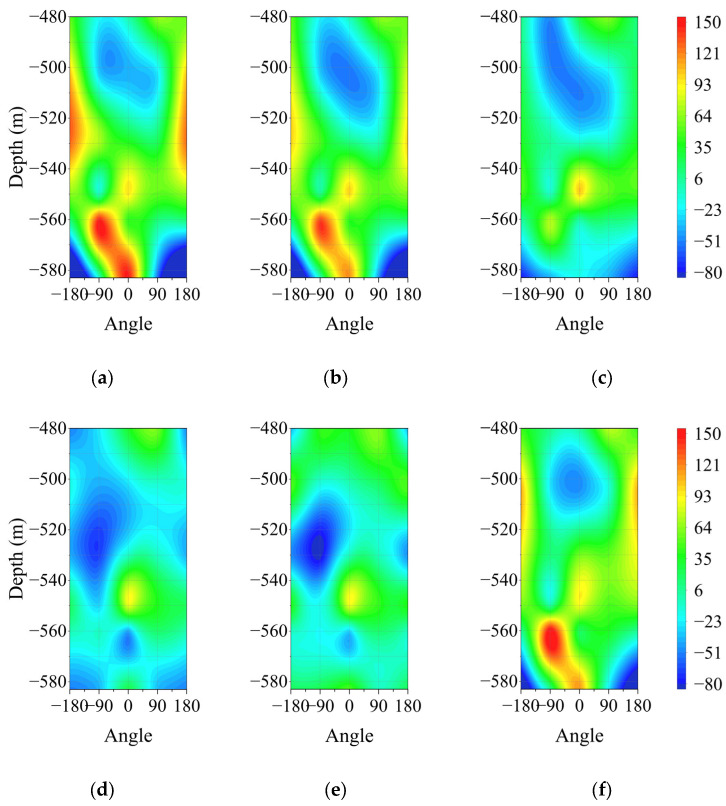
Contour map of the circumferential strain monitoring of the wellbore wall for each month: (**a**) January to February; (**b**) March to April; (**c**) May to June; (**d**) July to August; (**e**) September to October; (**f**) November to December.

**Figure 8:**
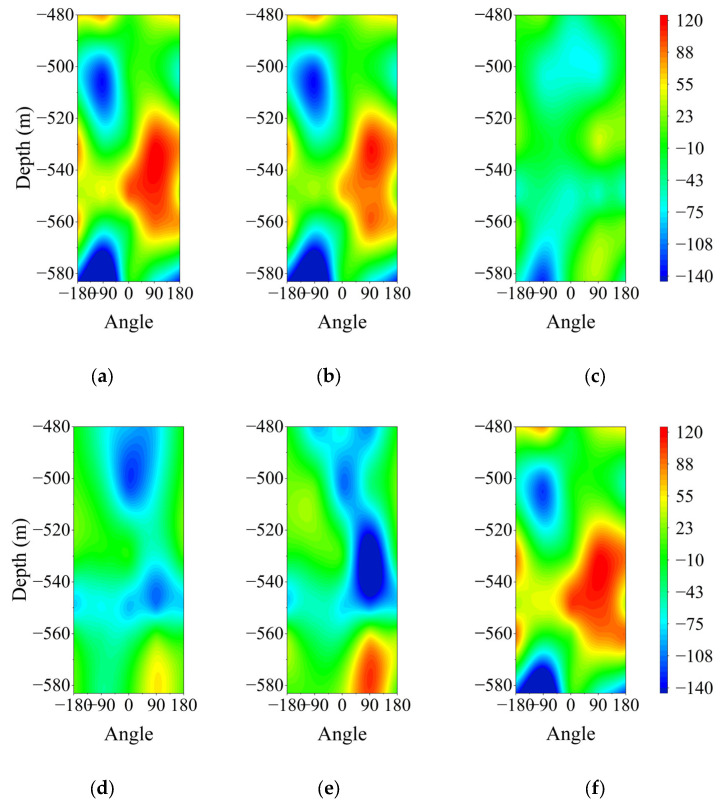
Contour map of the vertical strain monitoring of the wellbore wall for each month: (**a**) January to February; (**b**) March to April; (**c**) May to June; (**d**) July to August; (**e**) September to October; (**f**) November to December.

The strain distribution of the wellbore wall at various depths for each month is shown in the contour maps in Figure 7 and Figure 8. In these contour maps, the southeast direction is designated as 0°, while the wall is represented as a rectangular shape extending northwest. The horizontal coordinate of 90° indicates the southwest, and −90° indicates the northeast direction. The figures reveal that the strain distribution across all charts is generally consistent, allowing for the summarization of the circumferential and vertical strain variations with depth.

#### 4.1.1. Variation Law of Circumferential Strain with Depth

The strain values do not follow a simple linear trend with increasing depth. As illustrated in Figure 7 and Table 3, from the first to the sixth monitoring level, the extreme values of both circumferential compressive strain and circumferential tensile strain increased to approximately twice their original magnitudes. Tensile strain mainly occurs in the upper sections of the southeast and northeast directions. In contrast, compressive strain is prevalent in the lower sections. Conversely, in the southwest and northwest directions, compressive strain appears in the upper sections, while tensile strain occurs in the lower sections. The maximum tensile strain reaches 180.45 με at the fifth monitoring level in the northeast direction, while the maximum compressive strain is −98.97 με at the sixth monitoring level in the northwest direction. The circumferential strain is significantly influenced by groundwater seepage. At a depth of approximately 600 m, the sandstone within the wind-oxidized zone exhibits a water inflow rate of 0.894 L/(s·m) and a permeability coefficient ranging from 0.170 to 1.005 m/d. Mining activities that damage the coal seam floor contribute to water release from fractures in the sandstone.

The shaft, located on the western limb of the Juyi Syncline, features strata that incline from west to east at an angle of 5–10°. Additionally, the Bailihe and Tianqiao faults, coupled with pressure from the rigid rock layers to the east, produce a general trend of higher elevations to the west and lower elevations to the east. This results in a gentle slope to the southwest and a steeper slope to the northeast. Consequently, groundwater in the three aquifers flows from west to east along the strata, with more rapid seepage observed in the northeast direction. This pattern aligns with the strain trends indicated in the directional strain curves.

Furthermore, different stress conditions in the upper and lower sections of the shaft suggest that the soil mass on the east side of the upper section and the west side of the lower section exerts pressure on the shaft. This pressure causes the shaft, which is situated in a loose layer, to tilt slightly to the west. On-site measurements reveal that the greater volume of coal extraction to the northwest has weakened the lateral bending resistance of the shaft, making it likely to skew towards the mined-out area to the west. In the loose layer region, the shaft has already tilted approximately 20 mm, with more pronounced deformation observed at the shaft opening. Additionally, at the second monitoring level in the southwest direction, strain transitions from tensile strain to compressive strain. Conversely, at the fifth monitoring level in the northeast direction, strain shifts from compressive strain to tensile strain. This behavior can be attributed to two pressure relief notches near these levels on the wellbore wall. When the compressive device reaches maximum compression, cracks that form in the surrounding concrete tend to expand laterally, resulting in circumferential deformation of the shaft wall. This observation further supports the validity of the FBG monitoring results.

#### 4.1.2. Variation Law of Vertical Strain with Depth

The monitoring curves of vertical strain with depth variations, measured by multi-directional FBG sensors, are presented in Figure 8 and Table 4. The figure indicates that most orientations and depths exhibit tensile strain with considerable variation. Notably, significant changes occur at the compressible devices on the 2nd and 5th levels. The vertical strain on the borehole wall is primarily influenced by the vertical additional stress from the surrounding soil. This additional stress arises from a decrease in water levels in the aquifer below, causing soil settlement and deformation, which in turn creates pressure on the borehole wall. For example, the extreme values of vertical compressive strain in the southwest and northwest directions are both found at the 6th level, with values of −263.63 με and −141.93 με, respectively. This suggests that soil settlement in this area is pronounced and aligns with the trend of increasing additional stress with depth. However, substantial tensile strains also occur in all directions. The maximum tensile strain in the northwest direction is observed at the 4th level, measuring 120.96 με, while the northeast direction shows a maximum tensile strain of 146.22 με at the 3rd level. This variance is primarily related to the complex distribution of the surrounding rock. The borehole measures 882 m in length, consisting of a thin bedrock layer beneath a thick surface soil layer. The loose soil layer extends 583 m, whereas the bedrock layer is only 292 m thick, with weathered bedrock reaching a thickness of 115 m. The headgate, which connects the borehole to the tunnel, is positioned 184 m from the weathered bedrock zone. During tunnel construction, disturbances to the upper rock layers near the headgate are likely, leading to alterations in the stress state of the borehole wall and the generation of vertical tensile stress. Additionally, the western side of the borehole comprises a mined-out area, resulting in more significant changes in the overlying rock layers above the headgate and increasing the likelihood of tensile strain.

Moreover, the construction of the shaft is divided into two sections. The upper 702 m utilizes a freezing method to create a double-layer reinforced concrete wall, with concrete poured in segments. In contrast, the lower 180 m is built using a cast-in-place, single-layer plain concrete. There are variations in concrete grade and wall thickness between these two sections. Additionally, the reinforcing bars between segments are not connected through binding or welding. Consequently, vertical tensile stresses are transmitted upwards and released at the segment joints.

### 4.2. Relationship Between Strain and Date

#### 4.2.1. Variation Law of Circumferential Strain with Date

**Figure 9:**
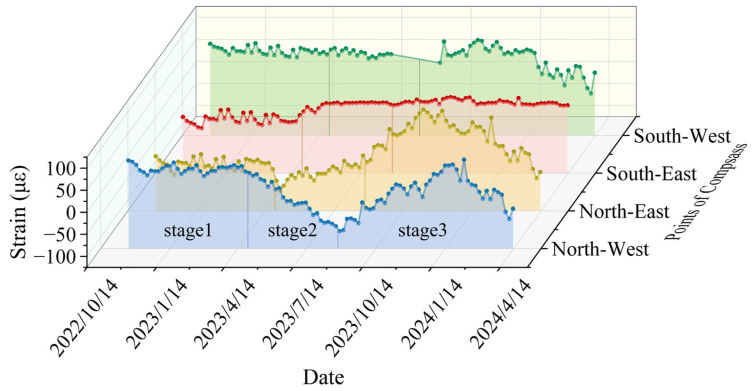
Circumferential strain versus date curve for the first monitoring level (cumulative depth of 481 m).

**Figure 10:**
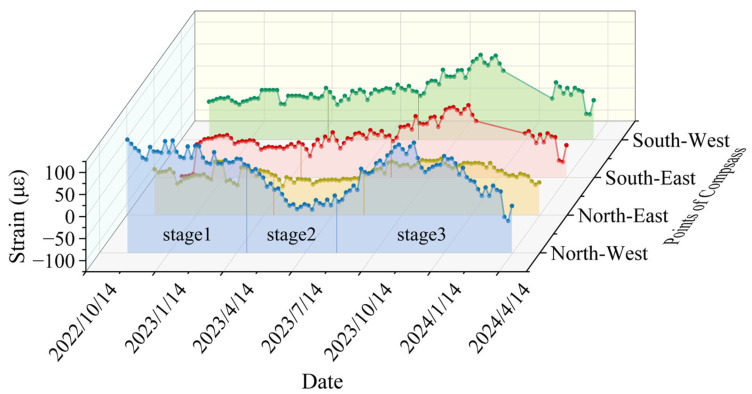
Circumferential strain versus date curve for the second monitoring level (cumulative depth of 503 m).

The radial strain versus time curves for two selected monitoring levels, out of six total levels, are presented in Figure 9 and Figure 10. In order to better interpret the temporal evolution of the shaft wall deformation, the monitoring period was divided into three stages based on the variations in monthly precipitation, air temperature, and groundwater recharge observed during the monitoring timeframe. The first stage, from 30 October 2022 to 30 March 2023, corresponds to the cold season with low precipitation and partial freezing of surface and shallow groundwater. During this period, groundwater recharge was minimal, and the additional stress acting on the shaft wall changed only slightly, resulting in relatively small strain variation rates The second stage, from 30 March 2023, to 30 August 2023, coincides with the main rainy season, during which intensive precipitation and increased river flow significantly enhanced groundwater recharge, leading to more significant strain variations, with a shift from compressive strain to tensile strain. The final stage, from 30 August 2023 to 30 January 2024, represents the post-rainfall period, characterized by decreasing precipitation and falling groundwater levels. This demonstrates a gradual decrease in compressive strain, followed by an increase in tensile strain, resulting in an overall upward curve. The rate of circumferential strain variation in the third stage is 37.58 με/month, which is approximately 1.48 times that in the first stage. This behavior is primarily influenced by local climatic conditions and groundwater effects on the wellbore. A detailed segment-by-segment analysis is as follows:

(1) The region has a temperate monsoon climate, with January being the coldest month, averaging −1.8 °C. During winter, surface and some groundwater freeze, limiting effective aquifer recharge and minimizing the soil layer’s impact on the wellbore. This explains the minor strain changes during the first stage. Furthermore, if the surrounding soil is highly water-saturated, freezing causes volume expansion that exerts pressure on the wellbore, resulting in compressive strain. For instance, during this stage, the maximum compressive strain at the first monitoring level reaches −79.65 με.
(2) The region experiences an average annual precipitation of 677.3 mm, primarily occurring in July and August, with a maximum daily rainfall of 223 mm. It also has an extensive network of artificial irrigation systems. During summer, abundant river flow replenishes groundwater, and some rainfall directly infiltrates the ground, leading to an increase in radial strain. The maximum strain at the first monitoring level during this period reaches 87.79 με. Higher summer temperatures, combined with increasing heat at greater depths, can cause concrete to expand and crack, further contributing to the rise in radial strain.
(3) After September, rainfall decreases and temperatures drop, which reduces aquifer recharge. This water loss leads to soil compression and deformation, while the weathered bedrock weakens the supporting strength of the overlying loose soil. Consequently, soil deformation intensifies and affects the wellbore more significantly. On 30 November, the maximum tensile strain at the first monitoring level reaches 72.79 με.

#### 4.2.2. Variation Law of Vertical Strain with Date

**Figure 11:**
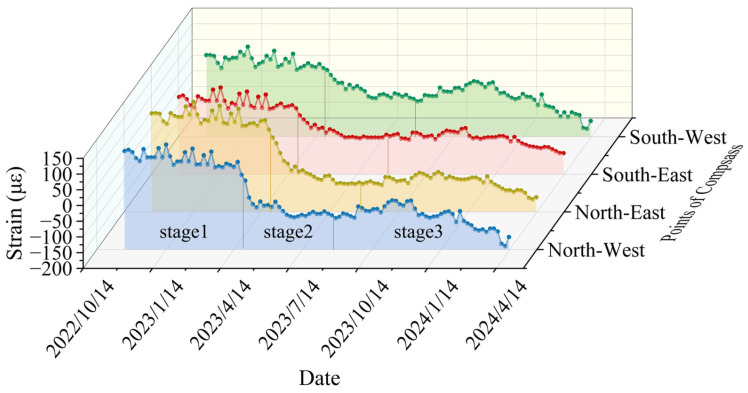
Vertical strain versus date curve for the fourth monitoring level (cumulative depth of 550 m).

**Figure 12:**
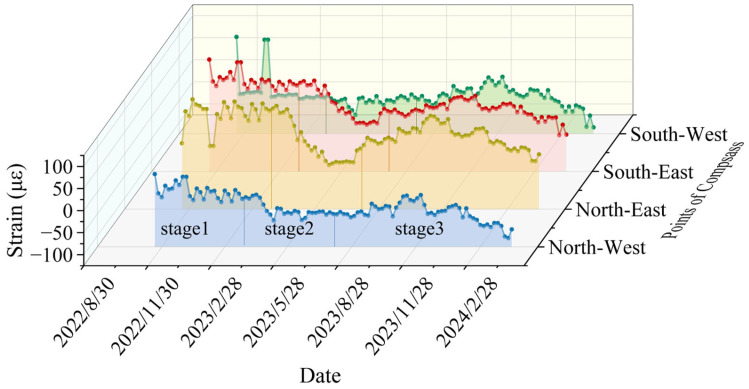
Vertical strain versus date curve for the fifth monitoring level (cumulative depth of 560 m).

The vertical strain versus time curves for two selected monitoring levels are presented in Figure 11 and Figure 12. The analysis method mirrors that used for radial strain over time, and the curve can also be divided into three stages. At the fourth monitoring level, the vertical strain exhibits a variation rate of 40.51 με/month in the third stage, representing an increase of about 42% relative to the first stage.

Stage 1 (30 October 2022 to 30 March 2023): During this stage, strain variation is minimal. Groundwater levels remain stable due to surface water freezing. Vertical additional stress is mainly influenced by the deformation of the overlying rock layers in the Maotoumen formation, which leads to tensile strain.

Stage 2 (30 March 2023 to 30 August 2023): In this stage, tensile strain gradually decreases and shifts to compressive strain, which exhibits slight fluctuations. This change results from increased summer precipitation, whereby groundwater flow in the aquifer increasingly dominates the additional stress over the deformation caused by the underlying bedrock, resulting in the development of compressive strain.

Stage 3 (30 August 2023 to 30 January 2024): The compressive strain continues to grow. During this phase, the soil begins to lose moisture and settle, increasing the negative frictional force on the wellbore, resulting in compressive vertical stress.

**Figure 13:**
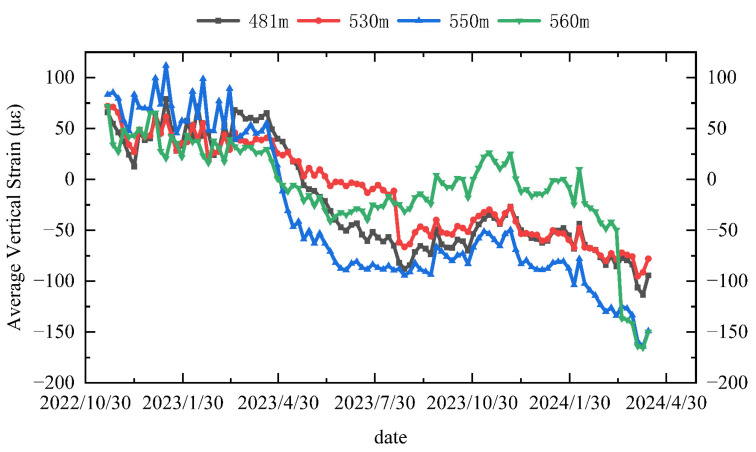
Long-term evolutionary temporal curves of the vertical average strain of the wellbore wall at different depth positions.

Additionally, to provide an overall indicator of the shaft wall’s deformation magnitude, an averaged strain curve was calculated by taking the arithmetic mean of the strains measured in the four circumferential directions. It should be emphasized that this averaged curve is not intended to replace or obscure the directional characteristics of the deformation. The anisotropic strain responses and directional differences have already been presented and analyzed in detail in the previous figures. The averaged curve in Figure 13 merely serves as a global trend representation, allowing a straightforward comparison of the general deformation level over time. The directional information remains fully preserved in the individual strain components, which constitute the primary basis for interpreting the mechanical behavior of the shaft lining. Consequently, the average strain curve for each layer was calculated by averaging the strains from these orientations, as shown in. The wellbore strain not only fluctuates with annual temperature variations but also exhibits a continuous cumulative trend. This indicates that the secondary wellbore experiences sustained compressive deformation from the downward movement of the industrial plaza’s strata. The additional force peaks at this depth, reflecting the highest strain accumulation. This deformation trend poses significant risks to the structural safety of the wellbore.

### 4.3. Analytical Solution of Wellbore Wall Stress

The hoop stress and vertical additional stress at each fiber optic grating sensor placement layer are calculated using the soil parameters from Table 2. The analysis shows that the undetermined constants, B*_i_*, are negligible and can be ignored. As a result, the displacement function is linear with respect to depth, *z*. The specific calculation results are presented in Table 5.

To further validate the analytical model of vertical additional stress and circumferential stress on the shaft lining, the stresses obtained from the analytical solution were compared with those derived from the measured strains at the corresponding monitoring levels. The measured circumferential and vertical strains were converted into stresses using the elastic modulus of the C70 and C50 concrete employed in the shaft lining, under the assumption of linear elastic behavior. As summarized in Table 6, both the analytical values and the measured values exhibit a consistent increasing trend with depth, reflecting the cumulative effect of overburden load and lateral pressure in deeper strata. The absolute differences between the analytical and measured stresses fall within a reasonable engineering range, which can be attributed to the simplifications made in the theoretical model, uncertainties in hydrogeological parameters, and local heterogeneity of the surrounding rock.

In addition, a comparison with the strength parameters and design criteria of the shaft lining concrete further confirms the safety of the structure. The standard compressive strength of C70 concrete under axial load is 44.5 MPa, while its tensile strength is 2.99 MPa. The peak vertical additional stress and circumferential stress obtained from both the analytical solution and the monitoring data remain significantly lower than the compressive and tensile strength values of the C70/C50 concrete, as well as below the allowable stress limits specified in the relevant standards. Therefore, the combined evidence from theoretical calculation, field monitoring, and material strength verification indicates that the shaft lining is currently operating within a safe stress range.

## 5. Conclusions

At the Guo Tun coal mine, six fiber optic grating sensors were installed at different depths to monitor hoop strain and vertical strain from four directions. This was done to establish a real-time monitoring system for shaft deformation, taking advantage of the high transmission accuracy and strong environmental adaptability of fiber optic grating sensors.

(1) The monitoring system successfully captured the spatial characteristics of shaft wall deformation. Both circumferential and vertical strains exhibit pronounced spatial anisotropy, with deeper monitoring levels showing significantly larger compressive and tensile deformation. The site-specific mechanical model that incorporates the stratigraphy, dual-layer concrete lining, and influence radius provides a clear physical basis for interpreting these deformation patterns.
(2) The temporal evolution of shaft wall strain is strongly influenced by groundwater variations and the consolidation of overlying strata. Quantitative analysis shows that strain increases progressively over time, consistent with the seasonal changes in groundwater recharge and subsequent drainage, demonstrating the importance of hydrogeological conditions in long-term deformation development.
(3) The stresses inferred from the measured strains agree well with the analytical solution in both magnitude and depth-dependent trend. All stress values remain below the strength limits of the C70/C50 concrete lining and the allowable values specified by relevant standards, indicating that the shaft is currently in a safe stress state. These results also validate the effectiveness of the proposed monitoring–analysis framework for evaluating shaft deformation under complex hydrogeological conditions.

## Figures and Tables

**Table 1:** Performance parameters of the FBG interrogator.

FBG Interrogator	Performance Parameters
Model	NZS-FBG-A02
Number of channels	8/16/32
Wavelength range (nm)	1527~1568
Wavelength resolution (pm)	1
Repeatability (pm)	±3
Demodulation rate (Hz)	1
Dynamic range (dB)	35
Optical interface type	FC/APC

**Table 2:** FBG installation horizontal layer arrangement.

Monitoring Layer	Depth (m)	Soil Properties	Layer Thickness (m)	Elastic Modulus (MPa)	Poisson’s Ratio *ν*	Unit Weight γ (kN/m^3^)
1	481	Fine Sand	22.7	25	0.32	20.0
2	503	Clay	26.6	14	0.29	18.9
3	530	Medium Sand	15.3	17	0.38	18.7
4	550	Medium Sand	12.5	25	0.33	18.7
5	560	Coarse Sand	6.7	35	0.30	18.8
6	583	Coarse Sand	18.4	48	0.25	18.2

**Table 3:** Extreme values of circumferential strain at each monitoring level.

Layer	Depth (m)	Maximum Circumferential Strain (με)	Minimum Circumferential Strain (με)
1	481	74.68	−51.50
2	503	109.43	−55.03
3	530	127.51	−68.46
4	550	100.82	−41.84
5	560	180.45	−44.48
6	583	148.52	−98.97

**Table 4:** Extreme values of vertical strain at each monitoring level.

Layer	Depth (m)	Maximum Circumferential Strain (με)	Minimum Circumferential Strain (με)
1	481	89.10	−102.18
2	503	18.61	−133.29
3	530	146.22	−191.06
4	550	120.96	−103.98
5	560	95.18	−50.01
6	583	102.25	−263.63

**Table 5:** Analytical solution for vertical and radial additional stress in the wellbore.

Monitoring Layer	Layer Thickness (m)	Influence Radius *R*_p_ (m)	Undetermined Coefficient *A*_i_ (10^−9^)	Vertical Additional Stress *σ*_z_’ (kPa)	Circumferential Strain *σ_θ_* (MPa)
1	22.7	171.19	10.2	4.09	−16.52
2	26.6	188.19	18.0	10.71	−17.28
3	15.3	169.07	5.06	15.81	−18.21
4	12.5	186.90	1.81	16.12	−18.90
5	6.7	197.61	0.32	20.16	−19.24
6	18.4	218.63	1.96	38.19	−20.03

**Table 6:** Comparison between the analytical and measured values at each monitoring level.

Monitoring Layer	Vertical Additional Stress σ_z_′ (kPa)		Circumferential Strain σ_θ_ (MPa)	
	Theoretical Value	Measured Value	Theoretical Value	Measured Value
1	4.09	7.52	−16.52	−20.32
2	10.71	14.28	−17.28	−22.57
3	15.81	17.17	−18.21	−22.95
4	16.12	20.43	−18.90	−24.13
5	20.16	25.86	−19.24	−24.88
6	38.19	30.26	−20.03	−26.42

## Data Availability

The authors do not have permission to share data due to the nature of this research; participants of this study did not agree for their data to be shared publicly.
